# Ballistic Gels in Experimental Fracture Setting

**DOI:** 10.3390/gels10070461

**Published:** 2024-07-14

**Authors:** Christoph Biehl, Ann-Cathrin Thiesse-Kraul, Sabine Stötzel, Salsabel Alzubi, Lotta Biehl, Matthias Mülke, Christian Heiss, Thaqif El Khassawna

**Affiliations:** 1 Department of Trauma, Hand and Reconstructive Surgery, Faculty of Medicine, Justus-Liebig-University of Giessen, 35392 Giessen, Germany; matthias.muelke@chiru.med.uni-giessen.de (M.M.);; 2 Experimental Trauma Surgery, Faculty of Medicine, Justus-Liebig-University of Giessen, 35392 Giessen, Germanythaqif.elkhassawna@chiru.med.uni-giessen.de (T.E.K.);; 3 Medical Faculty Heidelberg, Heidelberg University, 69117 Heidelberg, Germany; 4 School of Pharmacy, The University of Jordan, Amman 11942, Jordan

**Keywords:** ballistic gel, fracture model, pressure loads, biomechanical testing

## Abstract

Biomechanical tests typically involve bending, compression, or shear stress, while fall tests are less common. The main challenge in performing fall tests is the non-reproducible directionality of bone when tested with soft tissue. Upon removal of the soft tissue, the explanted bone’s resistance to impact diminishes. Therefore, ballistic gels can fix specimens in reproducible directions and simulate periosteal soft tissue. However, the use of ballistic gels in biomechanical studies is neither standardized nor widespread. This study aimed to optimize a ballistic gel consistency that mimics the upper thigh muscle in sheep. Our results suggest a standardized and flexible evaluation method by embedding samples in ballistic gel. Compression tests were conducted using cylindrical pieces of gluteal muscle from sheep. Various compositions of agarose and gelatin mixtures were tested to achieve a muscle-like consistency. The muscle-equivalent ballistic gel was found to consist of 29.5% gelatin and 0.35% agarose. Bones remained stable within the ballistic gel setup after freeze–thaw cycles between −20 °C and +20 °C. This method reduces the variability caused by muscle and improves storage quality, allowing for tests to be conducted under consistent conditionsBallistic gels of agarose and gelatin are suitable for bone fracture models. They have muscle-like strength, fix fractures simultaneously, are inexpensive to produce, and can be stored to allow repeated measurements of the same object with changing questions.

## 1. Introduction

Biomechanical testing is crucial in medical research related to human fractures, particularly in forensic medicine and military science, to study the directionality and efficacy of projectiles [1,2]. In experimental research setups, artificial bones (e.g., Sawbone^®^) are frequently used to establish baseline data and testing protocols during the initial phases. However, for more accurate and representative results that approximate living bone under physiological and pathological conditions, the use of biological bones from animal or human origins is necessary. These biological specimens provide more realistic responses due to their complex structure and composition. Recent research is increasingly focusing on the soft tissues, as these can absorb part of the energy and thus influence the effects on the bone [3,4]. Typically, biomechanical tests involve compression, flexure, or shear tests with permanently fixed objects without considering recapitulating the soft-tissue coverage [5]. These tests provide insights into the mechanical loading and failure characteristics of bones, implants, and their connections under repeated low-impact stresses, focusing primarily on material fatigue. However, such setups do not adequately simulate falls, which generally impose high-impact loads on localized areas, potentially causing complex fractures such as those of the proximal femur.

In forensic investigations of high-impact injuries, such as gunshot or stab wounds, bone fragmentation is recorded with high-speed cameras [6,7]. Compression or shear tests on non-embedded, thawed specimens are constrained in terms of procedural flexibility and temporal limitations. Embedding test objects in a ballistic gel of gelatin allows for spatial and temporal independence in evaluation, facilitating advanced imaging techniques such as X-ray, micro-CT, and MRI. This methodological improvement enables comprehensive ex vivo testing and data collection independent of the samples’ timing, storage, and age. Yet, reconstructing the dispersion of bone fragments within soft tissue remains challenging [2,8]. This necessitates additional testing using tissues embedded in ballistic gels. To make realistic assessments of the behavior of objects under accident conditions or indirect force, impact tests using pendulum or drop mechanisms are performed, requiring objects to be securely fixed. Ballistic gels, extensively used in forensic and military applications, provide a solution by simulating the mechanical properties of soft tissues under various testing conditions [9,10,11]. Depending on the test setup, these gels, made from agarose or gelatin, can secure projectiles and tissue fragments or simulate the strength of human tissues (e.g., brain, breast, blood vessels) [12]. Moreover, ballistic gels are employed in medical research as phantoms for internal organs, such as the lungs and prostate, facilitating standardized testing protocols [10,13,14]. The primary advantages of using ballistic gels over non-embedded native tissues include cost-effectiveness, versatility, reproducibility, remeasurement under controlled conditions, and ethical considerations [13,15,16]. Despite these benefits, the static mechanical properties of these gels often do not align with the dynamic requirements of medical biomechanical testing [17]. Ballistic gels often have a different, usually lower, modulus of elasticity than the tissue to be compared [18].

Experimental fracture models impose specific demands on the embedding medium. These settings rarely involve high-rate energy transfer, as seen in projectile impacts, or low-rate energy transfer, typical of mechanical interventions like surgical simulations [15].

Research has demonstrated that biological tissues exhibit different responses at high strain rates than at low strain rates [1,19,20]. Consequently, the behavior of native tissue under test conditions is non-linear, necessitating the use of ballistic gels that closely mimic the mechanical properties of the tissues being studied [21]. To accurately assess bony materials in drop tests, it is imperative to identify an appropriate medium to fix the test specimens. Traditionally, ballistic gels have been used to embed and fix specimens without replicating the mechanical strength of the periosteal structures. While this is often sufficient for many research questions, it is inadequate for fracture models where simulating the periarticular and periosteal tissues is crucial due to their significant influence on fracture mechanics [22]. Ballistic gels employed in drop tests should possess a Young’s modulus comparable to periosteal tissue [23]. Previous studies, such as the work by Van Sligtenhorst et al., have compared the elasticity of bovine muscle fibrils to that of gelatin [24]. Despite the potential benefits, the application of ballistic gels in biomechanical studies lacks standardization.

To test this hypothesis, we performed a series of compression tests on cylindrical samples of sheep gluteal muscle and compared the results with various ballistic gel compositions. The innovative aspect of this research is the establishment of a standardized, reproducible method for embedding bones in ballistic gel, thereby simulating the mechanical behavior of soft tissues during biomechanical tests. This approach also allows for consistent sample handling and storage, minimizing the variability caused by different handling techniques and storage conditions.

This study addresses critical gaps in previous research by enhancing the consistency and reproducibility of ballistic gels. It provides a practical embedding medium that replicates the mechanical properties of muscle tissue, facilitating more precise biomechanical testing and potentially increasing the reliability of experimental fracture models. Moreover, the findings of this study have broader implications for forensic and military science, where understanding tissue behavior under varying conditions is essential.

## 2. Results and Discussion 

A force of at least 400 N was calculated as necessary to break the proximal femur of the sheep in a drop test. To determine the properties of a suitable gel equivalent to the periosteal and muscular tissue, a compression test was used with a pressure-measuring device and an associated test program. A test series of 32 different gel combinations was conducted to identify the best match for simulating the periosteal structures.

First, ballistic gels of pure gelatin were produced and tested according to known protocols. Additionally, gels of pure agarose at different concentrations were tested. We found that gels with a composition of 0.35% agarose and 29.5% gelatin were comparable to the tested muscle tissue in terms of biomechanical requirements, showing values of 364 N compared with 399 N (*p* < 0.05).

**Figure 1:**
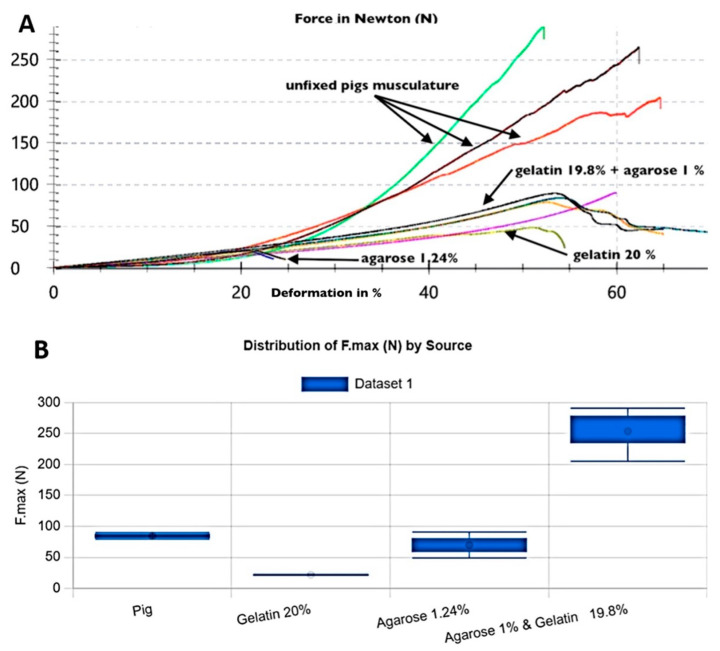
Maximum force (F.max) values achieved by different materials in biomechanical testing. This figure compares the performance of singular gelatin (20%), agarose (1.24%), and a mixture of gelatin (19.8%) and agarose (1.0%) against unfixed pig muscle samples. Pig muscle samples achieved higher F.max values (200–291 N) compared with the tested gels. Force-line testing single component ballistic gels vs. pig muscle (force max = 400 N). Sample-no 1 = 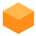, 2 = 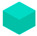, 3 = 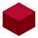, 4 = 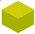, 5 = 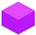, 6 = 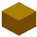, 7 = 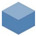, 8 = 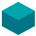, 9 = 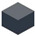, 10 = 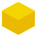.

Singular gels (Table 1, samples 4–5: gelatin 20%; samples 6–7: agarose 1.24%) were tested against the unfixed muscle of pigs (Figure 1). These muscle samples achieved values of 200–291 N, and thus did not reach the possible 400 N of the test apparatus. The singular gelatin or agarose gels, with an average maximum force of 90 N for gelatin and 22 N for agarose, also missed the required values. Some component gels of agarose and gelatin (samples 8–9: gelatin 19.8% + agarose 1.0%) showed better results than singular gelatin (max. 90 N) (Figure 1).

The statistical analysis provides a detailed comparison of the sources. Pig samples have the highest mean F.max (253.67 N) but the lowest mean L0 (12.81 mm). Agarose 1% and gelatine 19.8% has the highest mean L0 (25.6 mm) and the second-highest mean F.max (84.5 N). Gelatin 20% has a similar L0 to agarose 1% and gelatin 19.8%, but a lower F.max. Agarose 1.24% has the lowest mean F.max (21.95 N) and the second-lowest mean L0 (22.94 mm).

The ANOVA test shows a significant difference in F.max (N) among the different sources (F-statistic = 33.85, *p*-value = 0.00037), indicating that the maximum force (F.max) varies significantly depending on the source material. The scatter plot illustrates the relationship between L0 (mm) and F.max (N) for each source, while the box plot shows the distribution of F.max (N) for each source.

The low values were not based on a failure of the musculature, but on the destruction of the cell structures. In the red muscle tissue, there was no tissue tearing at room temperature. Rather, the tissue was crushed early, and the incorporated cell-bound fluid escaped. Veritable pressures could not be achieved in this way. By better fixing the muscle tissue and adjusting the force cut-off threshold to 400 N, comparability between gel and muscle could be achieved.

The testing machine was configured accordingly for the values (max. deformation, force cut-off threshold, upper force limit), and a test end was determined. When testing various muscle preparations, reaching the previously set upper force limit of 400 N led to the end of the test. (The maximum deformation of 50% (force cut-off threshold) was not reached here because the upper force limit had been reached previously). The gels of the test series were then tested with different compositions of agarose and gelatin. Gels with a high agarose content could not bind enough water, and therefore showed too little elasticity during testing. They burst before the end of the stamp test. If the gels had a high gelatin content, water was better bound in the gel, but the gels did not resist the stamp sufficiently and showed a “flow”. The test series showed an inverse proportional correlation between compressive strength and agarose content. With a lower agarose content, the compressive strength of the gel increased.

We found that gels with a 0.35% agarose and 29.5% gelatin composition were comparable to the tested muscle tissue for the biomechanical requirements (sample 1–3: 364 N acc. to sample 4–6: 399 N, testing temperature 17–19 °C, *p* < 0.05).

**Figure 2:**
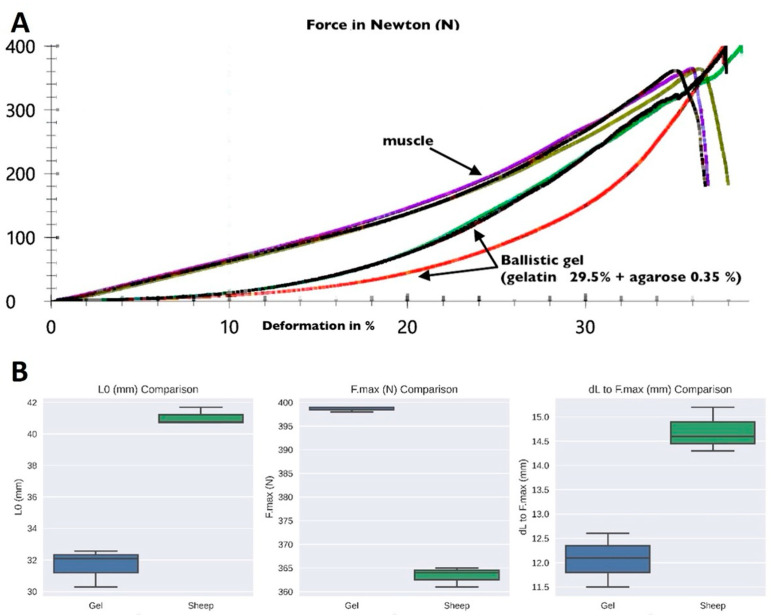
Force-line testing component ballistic gels (sample 4–6) vs. sheep muscle (sample 1–3) (force max = 400 N). Source-no: 1 = 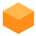, 2 = 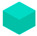, 3 = 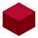, 4 = 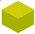, 5 = 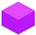, 6 = 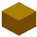.

During the testing of various gel preparations, reaching the previously set force cut-off threshold (50% of F max) or breaking the test object led to the end of the test (Table 2, Figure 2).

### 2.1. Results of Compression Measurements

The test series included singular gels with defined gelatin content (25%) and defined agarose content (1.25%). These were compared with combined gels (25% gelatin and 1.25% agarose) and porcine muscle tissue (Table 1 and Table 2). The musculature of sheep, which has a higher myoglobin concentration and more S-fibers than that of pigs, was also tested to simulate periosteal tissue realistically. Tests with non-fixed sheep muscle tissue were conducted under laboratory conditions and compared with agarose–gelatin gels (29.5% gelatin and 0.35% agarose). Preliminary tests with different concentrations of gelatin and agarose were performed to find the optimal composition for simulating sheep muscle tissue.

The musculature of sheep has a significantly higher myoglobin concentration with S-fibers than that of pigs. Because the periosteal tissue of sheep’s femora should be simulated as realistically as possible, further experiments were carried out with non-fixed muscle tissue from sheep. This was tested under laboratory conditions for compressive strength (samples 1–3) and compared with agarose-gel (samples 4–6; gelatin 42% and agarose 0.5%). To adapt the gels to sheep muscle, preliminary tests were performed with different concentrations of gelatin and agarose in small cylinders (<50 mL). Gels that did not pass the digital mechanical and optical tests were discarded. The gels with mixing ratios that appeared favorable were then further specified.

The results for periosseous muscle tissue were followed by a test series of ballistic gels with different ratios of gelatin and agarose (Table 2).

**Figure 3:**
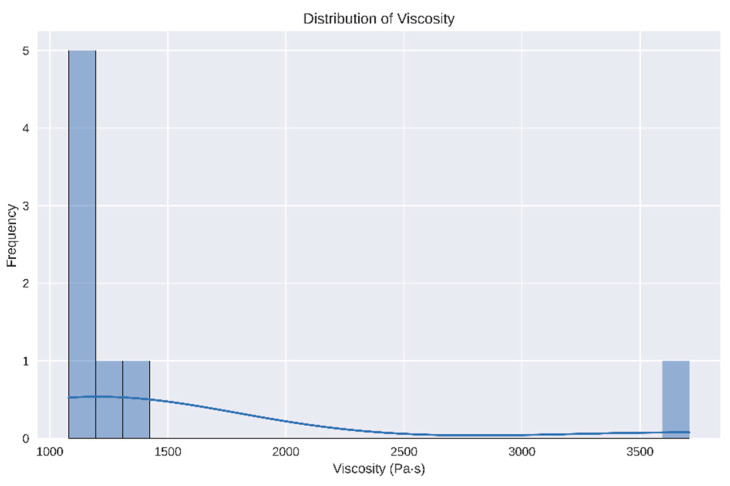
Histogram showing the distribution of viscosity values across all samples. The histogram indicates a right-skewed distribution, aligning with the results of the Shapiro–Wilk test for normality.

For use in the experimental setup, the ballistic gel was subjected to the usual cooling process and multiple changes between storage at −20 °C and investigations (X-ray, CT scan) at cooling temperatures (4–6 °C) and local heating by the energetic radiation in the CT. At the same time, the repeatability of the examination by X-ray and the optical control of the same femur at different times had to be ensured. The gel should not allow any changes to the object (Figure 3).

In this composition, ballistic gels with polysaccharides and proteins are comparable to periosseous tissue (musculature) and meet the requirements for biomechanical testing.

### 2.2. Rheology

We analyzed the rheological properties of the gel samples, focusing on viscosity and other related parameters. The viscosity measurements showed considerable variation across the samples, with a mean viscosity of 1517.485 Pa·s and a standard deviation of 890.16 Pa·s. The viscosity ranged from 1080.55 Pa·s to 3708.97 Pa·s. A Shapiro–Wilk test for normality yielded a *p*-value of 1.25 × 10^−5^, indicating that the viscosity data significantly deviates from a normal distribution (Figure 3). This non-normal distribution suggests the need for non-parametric statistical tests for further analysis.

Further analysis of the rheological properties revealed that the mean force was 62.19 N, with a standard deviation of 19.95 N and a range from 46.22 N to 109.64 N. The Shapiro–Wilk test *p*-value was 0.0015, indicating a deviation from normality. For displacement, the mean was 0.01 m, with a standard deviation of 0.00 m and a range from 0.00 m to 0.01 m. The Shapiro–Wilk test *p*-value of 0.7206 suggested a normal distribution. The syringe diameter had a mean of 0.01 m, with a standard deviation of 0.00 m and a range from 0.01 m to 0.01 m, with a Shapiro–Wilk test *p*-value of 1.0000, indicating normality. The mean flow rate was 0.00 m^3^/s, with a standard deviation of 0.00 m³/s, a range from 0.00 m^3^/s to 0.00 m^3^/s, and a Shapiro–Wilk test *p*-value of 0.6757, suggesting normal distribution.

The mean shear rate was 56.02 s^−1^, with a standard deviation of 9.72 s^−1^ and a range from 37.59 s^−1^ to 68.11 s^−1^, with a Shapiro–Wilk test *p*-value of 0.7137, indicating normality. For shear stress, the mean was 79,029.37 Pa with a standard deviation of 25,375.68 Pa and a range from 58,772.29 Pa to 139,406.98 Pa. The Shapiro–Wilk test *p*-value of 0.0015 indicated a deviation from normality. The viscosity had a mean of 1517.48 Pa·s, with a standard deviation of 890.16 Pa·s and a range from 1080.55 Pa·s to 3708.97 Pa·s, with a Shapiro–Wilk test *p*-value of 0.0000, indicating a non-normal distribution. The mean pressure was 791,795.84 Pa, with a standard deviation of 253,995.42 Pa and a range from 588,491.32 Pa to 1,395,979.84 Pa, with a Shapiro–Wilk test *p*-value of 0.0015, indicating a deviation from normality. The mean flow velocity was 0.01 m/s, with a standard deviation of 0.00 m/s and a range from 0.00 m/s to 0.01 m/s, with a Shapiro–Wilk test *p*-value of 0.6757, suggesting normal distribution.

These rheological properties provide essential insights into the material characteristics, which are crucial for further biomechanical applications. The viscosity measurements, in particular, displayed considerable variation, with a wide range and significant standard deviation. Most parameters showed a relatively normal distribution except for force, shear stress, viscosity, and pressure, which significantly deviated from normality.

**Figure 4:**
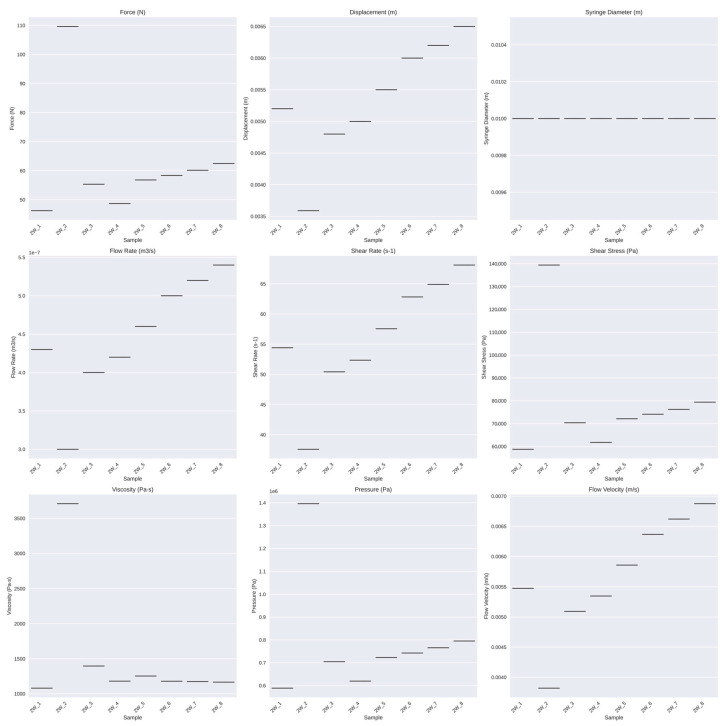
Box plots illustrating the distribution of each rheological property (force, displacement, syringe diameter, flow rate, shear rate, shear stress, viscosity, pressure, and flow velocity) across different samples. These plots help visualize the spread, central tendency, and potential outliers for each property.

These findings are visually confirmed by the box plots (Figure 4).

**Figure 5:**
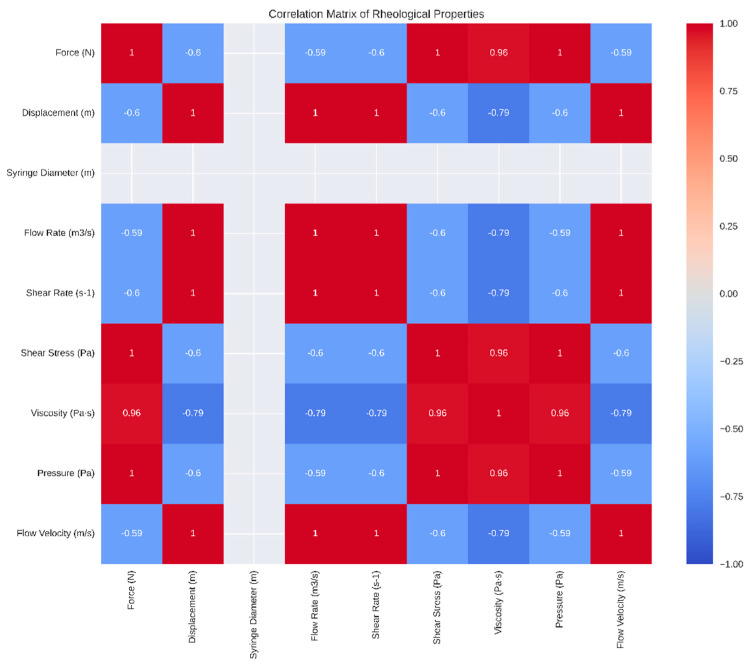
Heatmap displaying the correlations between different rheological properties. Stronger correlations are represented by darker colors, while weaker correlations are shown in lighter colors, providing insights into the relationships between the various properties.

The box plots show the distribution of each parameter across different samples, helping visualize the spread, central tendency, and potential outliers for each rheological property. The correlation matrix heatmap displays the correlations between different rheological properties, with stronger correlations represented by darker colors and weaker correlations by lighter colors (Figure 5). These visualizations offer additional insights into the relationships between the various rheological properties.

### 2.3. Discussion

In living organisms, bones are surrounded by periosteal tissue, which, depending on its arrangement and structure, prevents major dislocation of fragments in the event of a fracture. This tissue offers resistance to applied force similar to that of bone. According to Hill’s muscle model, which we detailed in the methodology section, the force of the contractile elements (FCE) can be calculated and combined with passive muscle force to estimate up to 60 Newtons per square centimeter of muscle area [25,26]. To test the fracture behavior of proximal femurs, the target area was defined as the trochanteric massif. At this point, the weight should result in high-impact contact with the test specimens, as regularly occurs during falls. With a sample diameter of 3 cm in the test machine, this results in a calculated value of around 430 N: *F_max_* (N) = 60 N/cm^2^ X π X r^2^, where r is the radius in centimeters. For a radius of 1.5 cm (because the diameter is 3 cm), this would result in: *F_max_* (N) = 60 N/cm^2^ X π X (1.5 cm)^2^ ≈ 425 N.

Consequently, the ballistic gel used should have a resistance similar to the test object, which in this study was the proximal femur. The impact area in the drop test was also around 3 cm in diameter. 

Biomechanical tests of bones are well-established and widely used in forensics and experimental medical research [26,27]. Most tests are performed on the diaphysis area using bending, pressure, or shear loads. However, pressure loads on the metaphysis or epiphysis are rare and occur only in special cases [28]. Clinically, fractures caused by falls are usually located in the metaphysis [29]. When simulating falls, drop tests provide a more realistic pathology compared with the static test procedures. The drop test setup developed for this study allows testing under realistic conditions. A significant challenge in such tests is the standardized reproducibility and fixation of samples.

To compare fracture models with patient data from human fractures, it is crucial to perform a comparable evaluation of high-impact trauma using micro-CT [30]. For an accurate assessment, fragments must be spatially fixed relative to the main fragment and surrounding soft tissue, necessitating the use of ballistic gels. Fracture models with preserved soft tissues (muscle, fat, skin) are realistic but have a limited study period due to decomposition processes [31]. Storage for later comparison is also restricted. Unfixed bones without soft tissues do not allow the spatial assignment of fragments and are unsuitable for these purposes. However, a non-fixed object is destroyed in this test setup. Ballistic gels offer the advantage over materials like sand or rice of permanently fixing fragments in space [32]. These alternative substances failed to provide adequate fixation, resulting in measurement inaccuracies due to the lack of enveloping structures necessary for subsequent image morphological evaluation using X-ray, MRI, or micro-CT [33].

Ballistic gels can be easily stored by freezing, allowing multiple analyses over time and for different research questions [34]. This capability facilitates the preservation of tissue samples obtained during euthanasia, aligning with the principles of the 3Rs (replacement, reduction, and refinement). However, the composition of the gels must be tailored to match the test objects [5]. Therefore, gels should be customized in terms of density, strength, and water binding to match the original periosteal soft tissue [35]. Ideally, gels could be fabricated from multiple layers with different compositions suited to specific tests [36]. Another challenge is the accurate positioning of specimens, because the area to be tested is not visible. Clear gels have the advantage over opaque shells, as they do not require X-ray positioning (fluoroscopy) for adjustment and alignment [37]. Ballistic gels are thus ideal for fracture morphology [16]. Depending on the requirements, various compositions of ballistic gels are known and used [2,17]. Combining fall simulation tests with fracture morphology has not been previously described. Embedding specimens in ballistic gels removes the time and location constraints of biomechanical testing, allowing for time-delayed or repeatable evaluations. Ex vivo tests with ballistic gels can be performed in animal experiments regardless of the time of euthanasia, thereby fulfilling the 3R principles.

Although gelatin gels bound sufficient liquid, they were suitable as the sole embedding medium only for small samples due to their low inherent stability. Agarose gels were more viscous but resulted in poorer fixation. Thus, developing a ballistic gel for the case studies that provided good specimen fixation with sufficient inherent stability was necessary. Both the biomechanical properties in the experiment and various evaluation settings confirmed the suitability of the combination of 0.35% agarose and 29.5% gelatin. The minor issues with gel residues remaining in the Erlenmeyer flask during production did not statistically affect the rheological properties and were irrelevant to the test questions.

### 2.4. Rheological Analysis Discussion

The rheological analysis of the gel samples aimed to evaluate the homogeneity and reproducibility of the gel mixtures, particularly in light of the observed remnants left in the flask after pouring the gel into molds. Despite these remnants, our analysis of eight samples demonstrated that the prepared gels maintained consistent rheological properties, indicating that the mixture was homogeneous.

The viscosity measurements showed considerable variation across samples, with a mean viscosity of 1517 Pa·s and a standard deviation of 890 Pa·s. The viscosity range from 1080 Pa·s to 3708 Pa·s suggests that the gel preparation process produces samples with a wide spectrum of flow behaviors. The Shapiro–Wilk test confirmed that the viscosity data deviated significantly from a normal distribution, highlighting the need for non-parametric statistical tests for further analysis.

Despite the observed remnants, the box plots illustrated that the rheological properties of the gels, including force, displacement, shear rate, shear stress, and flow velocity, were consistently distributed across the samples. This consistency suggests that the gel mixture achieved a high level of homogeneity, ensuring reliable and reproducible mechanical performance.

The correlation matrix heatmap further supported the uniformity of the gel properties, showing strong correlations between key rheological parameters. These findings confirm that the prepared gels are suitable for biomechanical applications, as they reliably replicate the mechanical behavior required for testing.

Overall, the rheological analysis indicates that the preparation method, despite some gel remnants in the flask, produces homogeneous and reproducible gel samples. This reliability is crucial for ensuring the validity of biomechanical tests using these gels, as it guarantees that the mechanical properties of the gels remain consistent across different samples. Future improvements could focus on minimizing the remnants left during the pouring process to further enhance the uniformity of the gel mixture.

### 2.5. Limitations

The study has several limitations. Firstly, the use of gelatin and agarose to prepare the ballistic gels was based on the authors’ considerations and not established laboratory protocols. Literature searches did not yield the expected results. Secondly, the number of test series to determine a gel density comparable to muscle was too low. It is hypothetical whether multiple tests would have led to different results and gel compositions. Thirdly, restricting the soft tissue to muscle only, without considering adipose tissue and skin, could limit the study. These choices were made for practical reasons. Moreover, specifying these ballistic gels was not the study’s original intent. The primary objective was to provide information on the fracture behavior of proximal femurs with varying bone densities (with and without osteoporosis).

## 3. Conclusions

Ballistic gels are used extensively in forensic and military applications to simulate the mechanical properties of biological tissues. For drop tests aimed at biomechanical research, it is crucial to accurately simulate the surrounding soft tissue to generate reliable and reproducible results. This study demonstrated that a combination of agarose and gelatin produces gels with mechanical properties closely resembling periosteal tissue, particularly muscle. The optimized gel composition of 0.35% agarose and 29.5% gelatin provides sufficient stability and elasticity to effectively mimic the resistance of periosteal and muscular tissues.

Moreover, the use of these ballistic gels allows for repeated evaluations without being constrained by time or location, facilitating comprehensive and versatile testing protocols. The ability to store and reuse samples through freezing further enhances the practicality and applicability of these gels in various experimental setups. By enabling consistent and accurate simulation of soft tissue, these ballistic gels support advanced biomechanical testing and contribute to the reduction of animal use in research, adhering to the principles of the 3Rs (replacement, reduction, and refinement).

## 4. Materials and Methods

Animal experiments were conducted in accordance with the Animal Welfare Act of the National Institute of Health and the guidelines for the care and use of laboratory animals. The experiments complied with the national animal welfare guidelines approved by the local regional government and conformed to German animal protection laws of the district government of Darmstadt (Ref. number V54—19c 20/15—F 31/36). All procedures were conducted at the central research facility of the Johann Wolfgang Goethe University in Frankfurt am Main. The study adhered to the guidelines of the Declaration of Helsinki and received approval from the Institutional Review Board (or Ethics Committee) (Ref. number 178/17).

### 4.1. Determination of Soft Tissue Properties

For testing osseous material in drop tests, a suitable medium for fixing the test objects was required. The embedding medium is needed to secure the object firmly while allowing the direct application of force. Additionally, it had to permit optical control and flexible evaluation regarding time and location. The medium also had to be simple, inexpensive to manufacture, and easy to dispose of. Ballistic gels, commonly used in medical settings, met these requirements. These gels are transparent, yellowish-tinged, and elastic. For this study, the ballistic gel was optimized to match the resistance of ordinary tissue.

Preliminary tests with muscle tissue from pigs and sheep and the determination of the corresponding components of the gels were performed under the same conditions as the intended tests (e.g., fracture settings under realistic temperature conditions).

**Figure 6:**
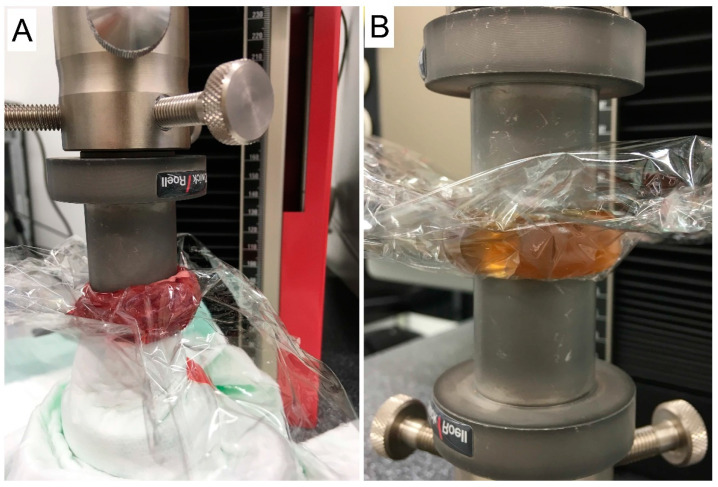
(**A**) Sheep muscle testing in the compression measuring device. (**B**) Testing ballistic gel in the compression measuring device.

Initially, muscle tissue from pigs was used as a reference to determine the mean values of the soft tissue surrounding the bone. Unfixed porcine thigh muscle with a specimen diameter of 3 cm was tested under compression. Sheep muscle has a significantly higher myoglobin concentration and more S-fibers than porcine muscle. Therefore, the test setup was repeated with sheep muscle to realistically simulate periosteal tissue (Figure 6A; Zwick Roell, zwickiLine 5 kN zwicki; software: testXpert III; Ulm, Germany).

The specimens were positioned so that the line of force during the compression test passed exactly through the center of the specimen. The specimens were preloaded with a force of 1 N. The test speed was 5 mm/min., and the maximum deformation was set to 50%. A measurement curve was generated at the end of the measurement.

This muscle tissue was tested (Figure 6A) for compressive strength under laboratory conditions and compared with different concentrations of agarose–gelatin gel.

### 4.2. Preparation and Testing of Ballistic Gels

Following the tests on muscle tissue, a series of tests were performed on ballistic gels with different concentrations of gelatin and agarose to match the properties of the gels to those of the muscle tissue (Figure 6B).

According to forensic protocols, preliminary tests were performed with gels of pure gelatin at different concentrations in small cylinders (<50 mL) (10% at 4 °C, according to the Fackler notes; 25% at 18 °C) (Table 2) [25,38,39]. Agarose gels were also tested. Gels that did not pass the digital mechanical and optical tests were discarded. The gels with a favorable mixing ratio were further specified and used for further testing.

The ballistic gels were prepared by dissolving 42% gelatin and 0.5% agarose in distilled water. The mixture was heated to 60 °C with constant stirring to ensure complete dissolution. Once completely dissolved, the solution was poured into molds to form cylindrical samples and cooled at room temperature. After cooling, the samples were stored at 4 °C until further use. Prior to testing, the gel cylinders were brought to room temperature. The prepared ballistic gel cylinders were subjected to compression tests using the same setup as the muscle tissue. The gels were molded into blocks 3 cm in diameter. Compression was applied at a 5 mm/min rate in the compression measuring device (Zwick Roell, zwickiLine 5 kN zwicki; software: testXpert III; Ulm, Germany) (Figure 6B).

By systematically testing and comparing the mechanical properties of muscle tissue and ballistic gels, we aimed to develop a standardized gel composition that closely mimics the mechanical behavior of periosteal tissue.

Following the muscle tissue tests, a series of tests were performed on ballistic gels with different concentrations of gelatin and agarose to approximate the properties of muscle tissue.

Specifically, agarose–gelatin gels were prepared and tested, with a 42% gelatin and 0.5% agarose concentration proving to be most similar to soft tissue.

### 4.3. Free-Fall Test Setup

**Figure 7:**
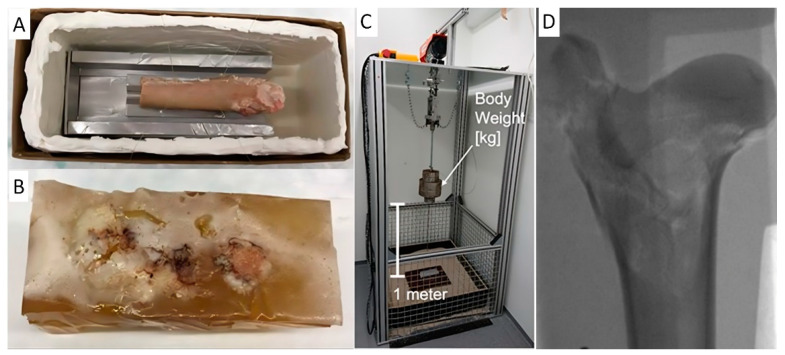
(**A**) Sheep femur pre-fixed at the sled prepared for pouring the gel. (**B**) Sheep femur covered in ballistic gel. (**C**) Free-fall tower device for free-fall testing. (**D**) X-ray of the proximal femur of the sheep, post-free-fall testing (Sheep K2).

In the free-fall test, high-impact trauma is simulated, the force of which is calculated using the formula F (force) = m (mass) × a (acceleration). For the subsequent test setup, the weight of the animals was measured at the time of euthanasia in the study and the drop weight was set accordingly (41–81 kg body weight. The standardized drop height in the study was 1 m) (Figure 7C). The acceleration was equal to the gravitational force g and was 9.81 m/s^2^. This resulted in an impact velocity on the trochanter region as follows:

Impact velocity = v(h) = √(2 × g × h) = √(2 × 9.81 m/s^2^ × 1 m) = 4.43 m/s = 15.95 km/h.

For casting the bones, 1000 mL was prepared; after pouring and cooling the gel overnight at 4 °C, it was allowed to acclimatize at room temperature for 4–6 h before testing (Figure 7A,B). The temperature was measured using an infrared thermometer to ensure consistency. Additionally, smaller quantities of 5–10 mL were tested as quality control to confirm the uniformity and consistency of the gel (Figure 6B).

### 4.4. Statistical Analysis

Statistical analyses were conducted using R to evaluate the mechanical and rheological properties of gel samples compared with biological tissues. Parameters such as force (F.max), initial length (L0), and deformation length to maximum force (dL to F.max) were measured. Descriptive statistics, including mean and standard deviation, were calculated for each parameter.

The Shapiro–Wilk test was used to assess the normality of data distributions, with a *p*-value < 0.05 indicating non-normality. One-way ANOVA was employed to determine significant differences in F.max among material sources (e.g., pig muscle, agarose–gelatin mixtures). For non-normally distributed data, non-parametric tests such as the Kruskal–Wallis test were used.

Additionally, statistical comparisons between the gel and sheep groups were conducted. The analysis included comparisons of initial length (L0), maximum force (F.max), and deformation length to maximum force (dL to F.max). Significant differences were assessed using *p*-values and effect sizes to determine the magnitude of the differences between groups.

The Hill muscle model was applied to calculate the force of the contractile elements (FCE). The formula used was FCE = Act × fim × fi × fvF, where Act represents the activation level, fim is the muscle’s intrinsic force, fi is the force interaction factor, and fv is the velocity factor. This model, combined with passive muscle force, allowed for the calculation of up to 60 Newtons per square centimeter of muscle area. This calculation was integrated into the evaluation of the mechanical properties to compare the resistance of the ballistic gel with biological tissues.

Rheological properties, including viscosity, were analyzed to understand material behavior under different conditions. Viscosity measurements were taken for the gel samples, and descriptive statistics were computed. The Shapiro–Wilk test checked for normality of viscosity data. Box plots visualized the distribution and variability of rheological properties across different samples, and a histogram showed the distribution of viscosity values. A correlation matrix heatmap illustrated relationships between different rheological properties, and correlation coefficients determined the strength and direction of these relationships.

All statistical analyses and visualizations were performed using R (version 4.0.5). Key packages included stats for conducting the Shapiro–Wilk test, ANOVA, and Kruskal–Wallis test; ggplot2 for creating box plots, histograms, and heatmaps; dplyr for data manipulation and summary statistics; and corrplot for generating the correlation matrix heatmap.

## Figures and Tables

**Table 1:** Testing ballistic gels (sample-no. 4–10) with singular component vs. pig muscles (sample-no. 1–3) (force max = 400 N).

Sample-no.	Source	L0 (mm)	F.max (N)	dL to F.max (mm)
1	Pig	14.38	205	9.3
2	Pig	10.6	291	5.5
3	Pig	13.45	265	8.4
4	Gelatin 20%	25.04	48.8	12.8
5	Gelatin 20%	25.07	90.7	15
6	Agarose 1.24%	21.85	21.4	4.6
7	Agarose 1.24%	24.04	22.5	4.9
8	Agarose 1% and gelatin 19.8%	25.25	84.3	13.6
9	Agarose 1% and gelatin 19.8%	25.87	89.9	13.8
10	Agarose 1% and gelatin 19.8%	25.68	79.3	13.5

**Table 2:** Testing ballistic gels with two components (sample-no. 1–3) vs. sheep muscle (sample-no. 4–6) (force max = 400 N).

Sample-No.	Source	L0 (mm)	F.max (N)	dL to F.max (mm)
1	Gel	32.09	399	12.1
2	Gel	32.57	399	12.6
3	Gel	30.29	398	11.5
4	Sheep	41.70	364	15.2
5	Sheep	40.71	365	14.6
6	Sheep	40.76	361	14.3

## Data Availability

The data supporting this study’s findings are available from the corresponding author upon reasonable request.
